# Metabolic regulation of obesity by naturally occurring compounds: mechanisms and therapeutic potential

**DOI:** 10.3389/fendo.2025.1655875

**Published:** 2025-09-12

**Authors:** Mousumi Das, Unnati Yagnik, Ishita Raninga, Debashis Banerjee

**Affiliations:** ^1^ Department of Life Sciences, Program – Microbiology, Atmiya University, Rajkot, Gujarat, India; ^2^ Departmentof LifeSciences, Program–Biotechnology, Atmiya University, Rajkot, Gujarat, India

**Keywords:** bioactive compounds, metabolic pathways, adipogenesis, lipolysis, gut microbiota, drug delivery

## Abstract

The global obesity epidemic continues to escalate, driving demands for safer and more effective therapeutic strategies. This review evaluates the potential of natural bioactive compounds as multi-targeted interventions for obesity management. Plant-derived polyphenols (e.g., epigallocatechin-3-gallate (EGCG), resveratrol), alkaloids (e.g., berberine), and marine carotenoids (e.g., fucoxanthin) demonstrate remarkable capacity to modulate fundamental obesity pathways through (A) suppression of adipogenesis via PPARγ and C/EBPα inhibition, (B) activation of lipolysis through hormone-sensitive lipase (HSL) stimulation and AMPK phosphorylation, (C) enhancement of thermogenesis via UCP1 upregulation, and (D) gut microbiome modulation through SCFA production. Clinical evidence supports the efficacy of selected compounds, with green tea catechins showing 4% to 5% body fat reduction and berberine demonstrating significant metabolic improvements. These natural agents offer distinct advantages over conventional drugs through their pleiotropic mechanisms and favorable safety profiles. However, bioavailability limitations and inter-individual variability present significant challenges. Innovative delivery systems, including nanoencapsulation and phospholipid complexes, show promise to enhance the therapeutic potential. The review highlights emerging strategies combining microbiome modulation with precision nutrition approaches while emphasizing the need for standardized clinical protocols. By bridging ethnopharmacological knowledge with modern scientific validation, natural compounds represent a promising avenue to develop sustainable, multi-targeted anti-obesity therapies that address both physiological and metabolic aspects of this complex disorder.

## Introduction

The global obesity epidemic represents one of the most significant public health crises of our time, with its prevalence having escalated dramatically across both developed and developing nations. Characterized by abnormal or excessive fat accumulation that impairs health, obesity significantly elevates the risk of numerous chronic diseases including type 2 diabetes, cardiovascular disorders, and certain cancers. Global obesity rates continue to rise alarmingly, with the World Health Organization (1) reporting that 1.9 billion adults are overweight, including 650 million with obesity. The International Diabetes Federation (2) highlights that obesity-driven type 2 diabetes now affects 537 million adults, with projections reaching 783 million by 2045. Case studies reveal regional disparities—Mexico’s 2024 national survey showed 75% adult overweight/obesity rates linked to ultra-processed food consumption (3), while Singapore’s health interventions reduced the childhood obesity by 5% through sugar tax and school programs (4). These trends underscore the urgent need for policy-driven solutions. Conventional treatment strategies focusing on lifestyle modifications and pharmaceutical interventions have demonstrated limited long-term success, plagued by issues of poor compliance, adverse side effects, and diminishing effectiveness over time. This therapeutic gap has spurred a growing scientific interest in exploring nature-derived bioactive compounds as potential alternatives or adjuncts in obesity management. Plants, marine organisms, and traditional medicinal herbs contain a diverse array of phytochemicals—including flavonoids, alkaloids, terpenoids, and polysaccharides—that exhibit multi-targeted anti-obesity properties. These natural agents work through complex mechanisms involving (A) the inhibition of adipocyte differentiation and lipid accumulation, (B) the enhancement of fat mobilization and energy expenditure, (C) the regulation of appetite and satiety signals, and (D) the modulation of gut microbiota composition and function. Particular attention has focused on compounds like epigallocatechin gallate (EGCG) from green tea, curcumin from turmeric, and capsaicin from chili peppers, which have shown promising metabolic benefits in preclinical and clinical studies. Their pleiotropic actions on molecular targets such as PPARs, AMPK, and uncoupling proteins offer distinct advantages over single-target pharmaceutical approaches (5). However, critical challenges including variable bioavailability, dose optimization, and standardization of bioactive components must be addressed to fully realize their therapeutic potential. This comprehensive review examines the scientific evidence supporting natural anti-obesity compounds, elucidates their mechanisms of action at the molecular and systemic levels, and discusses innovative strategies to overcome current limitations in their clinical application. By bridging traditional ethnopharmacological knowledge with cutting-edge biomedical research, these natural therapeutics may provide far more sustainable solutions to combat the global obesity pandemic. This analysis systematically examines the therapeutic mechanisms and clinical applications of bioactive phytochemicals for weight management while exploring their emerging role in personalized nutritional interventions. The review assesses molecular pathways targeted by these compounds, evaluates evidence from human trials, and discusses innovative strategies to implement them in precision-based approaches to metabolic health.

## Methodology

In order to present a thorough synthesis of preclinical and clinical data pertaining to the anti-obesity potential of naturally occurring bioactive chemicals, this review uses a narrative method enhanced by structured literature searches. The system, which focused on mechanistic discoveries and therapeutic relevance, identified pertinent studies across cellular, animal, and human models using an open and repeatable procedure.

### Literature search strategy

**Figure 1:**
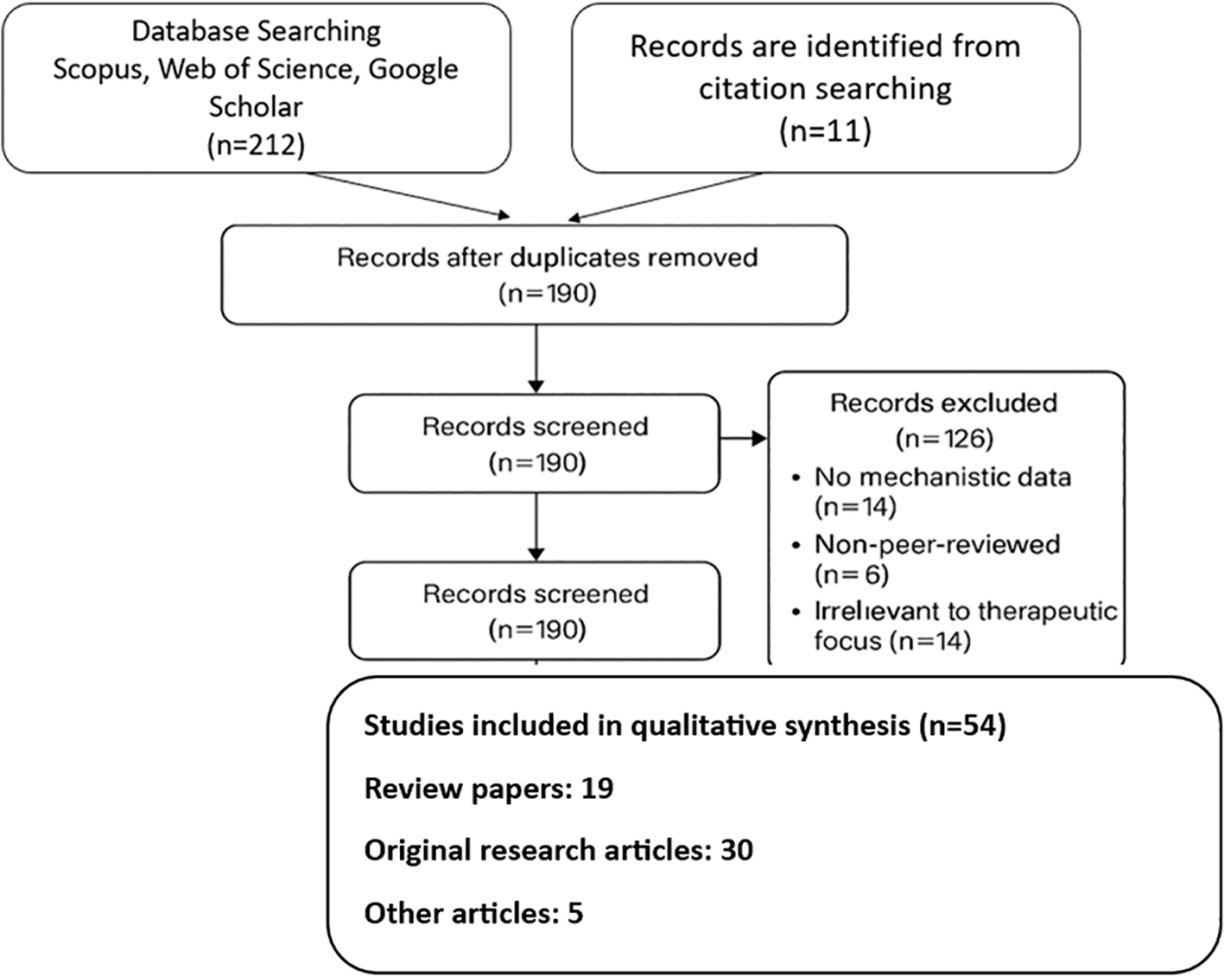
PRISMA chart showing the strategy of reviewing the data with regard to natural compounds and their role in therapeutic applications pertaining to obesity.

For research published between January 2000 and April 2024, a comprehensive literature search was conducted using the electronic databases PubMed, Scopus, Web of Science, and Google Scholar. The key terms and their Boolean combinations were “natural compounds” OR “bioactive compounds”, “obesity” OR “anti-obesity”, “adipogenesis” OR “lipolysis” OR “thermogenesis”, “polyphenols” OR “alkaloids” OR “carotenoids”, “green tea catechins” OR “EGCG” OR “resveratrol” OR “berberine” OR “fucoxanthin”, “gut microbiota” OR “microbiome modulation”, “drug delivery” OR “nanoencapsulation” OR “phospholipid complexes”. Only articles in the English language were taken into account. To further find important materials, the reference lists of chosen research and pertinent review articles were also manually screened (**Figure 1**).

### Inclusion and exclusion criteria

Original research presenting clinical data on the anti-obesity properties of natural substances in humans, animals, or *in vitro* models was one of the study’s inclusion criteria. Additionally taken into consideration were studies that clarified the molecular mechanisms of action, such as those involving the AMPK, PPARγ, and UCP1 pathways. Relevant reviews and meta-analyses that concentrated on distribution strategies or treatment results were also incorporated. On the other hand, the exclusion criteria excluded studies that focused exclusively on synthetic compounds, editorials and commentaries that were not subjected to peer review, and articles that lacked mechanistic insights.

### Data extraction and synthesis

Data from the chosen articles were methodically taken out and arranged by the class and source of natural compounds, including carotenoids, polyphenols, and alkaloids. There was also documentation of the experimental models used, which included clinical trials, animal models, and cell lines. Alongside the reported results, such as changes in body weight, fat mass, and metabolic parameters, mechanisms of action were identified, including the inhibition of adipogenesis, stimulation of thermogenesis, and modulation of gut microbiota. Additionally mentioned were delivery methods such as nano formulations and improvements in bioavailability. In order to identify convergent biological pathways and evaluate their translational potential, the synthesis combines qualitative and mechanistic findings. Clinical efficacy results were compiled where appropriate to highlight the natural compounds’ therapeutic value.

### Key metabolic pathways targeted by natural anti-obesity compounds

The prime focus in this area for the review is given on two important mechanisms like adipogenesis and lipogenesis.

### Adipogenesis and lipogenesis inhibition

The various mechanisms, when discussed, can take on the role of an enzyme.

### Target enzymes

The following descriptions provide a detailed overview on the role of natural anti-obesity compounds that often inhibit key enzymes involved in adipogenesis (fat cell formation) and lipogenesis (fat synthesis). The key targets include the following:

- Acetyl-CoA carboxylase (ACC): A rate-limiting enzyme in fatty acid synthesis, ACC converts acetyl-CoA to malonyl-CoA. Compounds like berberine and flavonoids suppress ACC activity, reducing fat accumulation.
- Fatty acid synthase (FAS): FAS catalyzes the synthesis of long-chain fatty acids. Polyphenols (e.g., resveratrol) and terpenoids inhibit FAS, disrupting lipid storage.

### Mechanisms

The following path can be discussed to explain the role of FAS. Obesity results from excessive fat accumulation due to dysregulated adipogenesis (formation of new fat cells) and lipogenesis (synthesis of fatty acids) (6).

Naturally occurring compounds target these processes through multiple mechanisms, namely:

#### Adipogenesis inhibition

Adipogenesis is controlled by a transcriptional cascade that converts preadipocytes into mature adipocytes. The ley factors and their inhibition by natural compounds include the following:

##### Transcriptional regulation

•PPARγ (peroxisome proliferator-activated receptor gamma): The peroxisome proliferator- activated receptor gamma plays an important role as master regulator of adipocyte differentiation. Various natural inhibitors like resveratrol (grapes, berries) ↓ (downregulates) PPARγ expression via SIRT1 activation. Quercetin (apples, onions) blocks PPARγ phosphorylation (7). C/EBPα (CCAAT/enhancer-binding protein alpha) works synergistically with PPARγ to promote adipocyte maturation. EGCG (green tea) suppresses C/EBPα via ERK pathway inhibition.

##### Early-stage adipogenic signals

• The following parameters in this regard can be discussed in brief as follows: The pathway, like Wnt/β-catenin pathway, follows in the stages as enlisted antiadipogenic’ its suppression promotes fat cell formation; berberine (goldenseal) activates Wnt signaling, blocking adipogenesis (8). One of the significant events that can be discussed over here is AMPK activation; metformin-like botanicals (e.g., galegine from goat’s rue) activate AMPK, inhibiting PPARγ.

### Lipogenesis inhibition

Lipogenesis involves *de novo* fatty acid synthesis, primarily in liver and adipose tissue. The key targets discussed in the following **Tables 1** and **2** describe the role of enzymes and natural compounds.

**Table 1:** Role of enzymes in action for the effective management of natural compounds for disorder management practices.

Enzyme	Role	Natural inhibitors	Mechanism	References
FAS (fatty acid synthase)	Synthesizes palmitate	Curcumin (turmeric)	Binds to ketoacyl synthase domain	(9)
SCD-1 (stearoyl- CoA desaturase-1)	Converts saturated → unsaturated fats	Genistein (soy)	Downregulates SCD-1 mRNA	(10)

**Table 2:** Natural obesity compounds and target key pathways.

Compound	Source	Target enzyme	Hormone modulated	Mechanism	References
Berberine	Golden seal	ACC	Adiponectin↑	AMPK activation→ ACC phosphorylation; ↑fatty acid oxidation	(25)
Curcumin	Turmeric	FAS, SCD-1	Leptin↑	Binds FAS active site; ↓SREBP-1c maturation; ↑leptin sensitivity	(9)
EGCG	Green tea	SCD-1	Insulin↓	↓SCD-1 via miR- 34a; improves insulin signaling	(24)
Resveratrol	Grapes	SIRT1	GLP-1↑	Activates SIRT1→ ↓PPARγ; ↑GLP-1 secretion	(47)
Fucoxanthin	Brown seaweed	PPARγ, ACC	Irisin↑	↑UCP1 in fat; irisin-mediated thermogenesis	(46)

↑, increase/upregulation; ↓, decrease/downregulation; →, “leads to” or “results in”; -, uncoupling protein 1 (UCP1) (thermogenic).

**Table 1** elaborates on the mechanism behind different metabolic reactions employing enzymes, which acts on natural inhibitors and promote the therapeutic insights by different mechanisms as cited in different research reports, respectively. The relatively underexplored challenges and delivery strategies for the clinical translation of curcumin and resveratrol have been addressed, with support from select literature reports. Key challenges include poor bioavailability, as both compounds exhibit low solubility, rapid metabolism, and fast systemic clearance, which collectively limit their therapeutic efficacy (11, 12). Chemical instability further hampers their potential, with curcumin degrading at physiological pH and resveratrol undergoing oxidation and isomerization (13). Additionally, dose-limiting toxicity has been reported, wherein high doses of resveratrol may induce gastrointestinal distress and excessive curcumin intake may lead to hepatotoxicity (14).

Several delivery approaches have been developed to overcome these barriers. Nanocarriers, such as liposomes, polymeric nanoparticles, and micelles, improve solubility and prolong systemic circulation (15, 16). Phospholipid complexes—for instance, Meriva^®^ for curcumin—enhance intestinal absorption (17). PEGylation and cyclodextrin inclusion increase stability and bioavailability (18), while prodrugs and chemical conjugates reduce metabolic degradation (19). Optimizing these delivery strategies is essential to fully harness the therapeutic potential of curcumin and resveratrol in clinical settings. **Table 2** presents the data for natural compounds employed in modulating hormone actions and influencing the mechanisms as described in various research reports.

Another mechanism for controlling transcriptional events will also be discussed as follows where the role of various compounds in genetic and physiological regulations as well metabolism is also described.

### Transcriptional control

The various control mechanisms are reviewed in research reports, and a few representative ones are stated here—for example, sterol regulatory element-binding protein 1c (SREBP-1c) and master regulator of lipogenic genes (ACC, FAS); Omega-3s (EPA/DHA) promote SREBP-1c proteasomal degradation. Another mechanism in fat storage is also described, like combined effects on fat storage. The various natural compounds often exhibit dual inhibition—for example, fucoxanthin (brown seaweed)↓ (downregulation) PPARγ and ↑ (upregulation) AMPK (leads to or results in) → reduces lipid droplet size by 60% in human adipocytes. The recent literature survey also enlightens in the section “Clinical Implications and Challenges” in research on the gap of the avenue to be explored in later stages in a similar line of action. They are as detailed in the following discussion.

### Synergy issues

While single compounds show efficacy, combinations (e.g., resveratrol + quercetin) require dose optimization to avoid antagonism. Facets on sex differences are also studied in this research. PPARγ inhibition is 23% more effective in female adipocytes. Despite the growing recognition of sex-based differences in PPARγ inhibition, several key challenges hinder progress in this field. First, biological complexity makes it difficult to isolate hormonal effects from other sex-specific factors, as estrogen’s interaction with PPARγ is influenced by metabolic, genetic, and epigenetic variables (20). Second, historical bias in preclinical and clinical research—particularly the underrepresentation of female subjects in early drug development—has led to a lack of sex-disaggregated data, obscuring true efficacy and safety differences (21). Third, hormonal variability in females (e.g., menstrual cycles, menopause, pregnancy) complicates the standardization of dosing regimens, raising concerns about treatment consistency (22). Additionally, regulatory and cultural barriers persist, as sex-specific drug development remains underprioritized due to higher costs and logistical challenges in trial design. Addressing these issues requires a multidisciplinary approach, including mandatory sex-balanced trials, advanced hormone-modulated delivery systems, and translational studies to bridge preclinical findings with clinical outcomes.

### Delivery advances

Nanoparticle-encapsulated EGCG improves adipose targeting by fourfold. A consolidated overview of the mechanism of action mediated by natural compounds to combat obesity issues is shown in **Table 3**. The tables also aims to focus on compounds that act on different obesity problems by targeting specific metabolic systems and key enzymes so that the target specific therapy or natural inhibition can be initiated. The focus should be on energy balance toward fat utilization rather than storage.

**Table 3:** Key metabolic pathways targeted by natural anti-obesity compounds.

Pathway	Target enzymes	Natural compounds	Mechanism of action	References
Adipogenesis inhibition	PPARγ, C/EBPα	Resveratrol, quercetin, EGCG	Downregulates adipogenic transcription factors, reducing fat cell formation	(23, 24)
Lipogenesis inhibition	Acetyl-CoA carboxylase (ACC)	Berberine, flavonoids	Inhibits ACC, blocking malonyl-CoA production and fatty acid synthesis	(25)
Triglyceride inhibition	Fatty acid synthase (FAS)	Curcumin, genistein	Suppresses FAS activity, reducing triglyceride accumulation	(26)
Lipolysis activation	Hormone-sensitive lipase (HSL)	Capsaicin, catechins	Stimulates HSL, enhancing the breakdown of stored triglycerides into free fatty acids	(27)
Fatty acid oxidation	Carnitine palmitoyl transferase-1 (CPT-1)	Omega-3s, genistein	Upregulates CPT-1, promoting mitochondrial β-oxidation and energy expenditure	(28)
Lipogenesis inhibition	AMP-activated protein kinase (AMPK)	Berberine, EGCG	Activates AMPK, inhibiting lipogenesis while stimulating fat oxidation	(29)

### Lipid metabolism enhancement

Natural compounds also enhance lipid breakdown by the following mechanism of targets:

- Hormone-sensitive lipase (HSL): This mechanism is found to be critical for triglyceride hydrolysis in adipocytes. Capsaicin (from chili peppers) and catechins stimulate HSL, promoting fat mobilization (30).
- Carnitine palmitoyltransferase-1 (CPT-1): The mechanism facilitates fatty acid oxidation in the mitochondria. Genistein (soy isoflavone) upregulates CPT-1, boosting energy expenditure (31).

A brief insight on the detailed mechanism has been elaborated as follows: activation of PPARα and PGC-1α increases mitochondrial fatty acid oxidation. Compounds like curcumin and omega-3s enhance these pathways‘ shifting.

### Promotion of lipolysis and thermogenesis

Target enzymes/proteins: The listed target enzymes/proteins play an important role in metabolic functions and inhibitory mechanisms, like HSL (hormone-sensitive lipase) and ATGL (adipose triglyceride lipase) which play a prominent role in the hydrolysis of triglycerides into free fatty acids.

Natural activators with their roles are enlisted as follow: capsaicin (chili peppers)–stimulates catecholamine release, activating HSL (32). Berberine (golden seal) upregulates AMPK, enhancing lipolysis (33). UCP1 (uncoupling protein 1): this important protein plays a vital role in mediating thermogenesis in brown adipose tissue (BAT). Natural inducers like common dietary and daily commodity things are also utilized as in the following examples: ginsenosides (ginseng)—activate UCP1 via SIRT1/PGC-1α pathway; caffeine (coffee, tea) —enhances BAT activity via β-adrenergic stimulation (34). Appetite regulation and satiety enhancement: In this regard, few important pathways were shared to understand the role of a few natural compounds.

### Target pathways

Leptin and ghrelin signaling: Leptin suppresses appetite (35), while ghrelin stimulates hunger.

Natural modulators: Various natural modulators can be effective in playing a significant role, and a few of them are described in detail as follows:

Fucoxanthin (brown seaweed)—This compound increases adiponectin, improving leptin sensitivity. Fiber (e.g., β-glucan, glucomannan), like compounds, delay gastric emptying, reducing ghrelin secretion. Likewise in the case of serotonin (5-HT) and dopamine pathways, the regulation of mood and food cravings was being observed. The natural modulators play a significant role by the following manner—for example, 5-HTP (*Griffonia simplicifolia*), a precursor to serotonin, reduces emotional eating, while L-theanine (green tea) used to modulate dopamine and decreases reward-driven eating.

### Gut microbiome modulation

Natural compounds, such as polyphenols (36), dietary fibers (37), and plant-derived bioactive molecules, modulate the gut microbiome to combat obesity through multiple mechanisms. These compounds enhance beneficial bacteria (e.g., *Akkermansia muciniphila*, *Bifidobacterium* spp.) while suppressing obesity-linked microbes (e.g., *Firmicutes*). They promote the production of short-chain fatty acids (SCFAs) like butyrate, which improve insulin sensitivity, reduce inflammation, and enhance satiety. Additionally, natural compounds inhibit endotoxin-producing bacteria, lowering metabolic endotoxemia and adipose tissue inflammation. By restoring microbial balance and improving gut barrier integrity, these bioactive molecules help regulate lipid metabolism and energy expenditure, offering a promising dietary strategy for obesity management through microbiome-targeted interventions. Emerging evidence highlights the crucial role of gut microbiota in modulating the efficacy of bioactive compounds through species-specific metabolic transformations. Studies demonstrate that gut bacteria convert compounds like curcumin and resveratrol into active metabolites (38, 39), while microbial-derived short-chain fatty acids influence host targets like PPARγ (40). The microbiome also affects drug bioavailability, with significant interpersonal variability observed (41). Specific bacterial taxa are implicated in polyphenol activation (42), and gnotobiotic models confirm causal relationships. However, establishing definitive microbe–compound interactions requires further mechanistic studies accounting for individual microbiome variations and standardized intervention protocols. These findings underscore the microbiome’s potential as a modifier of therapeutic outcomes. The mechanism will be described in the following discussion.

### Mechanisms

**Figure 2:**
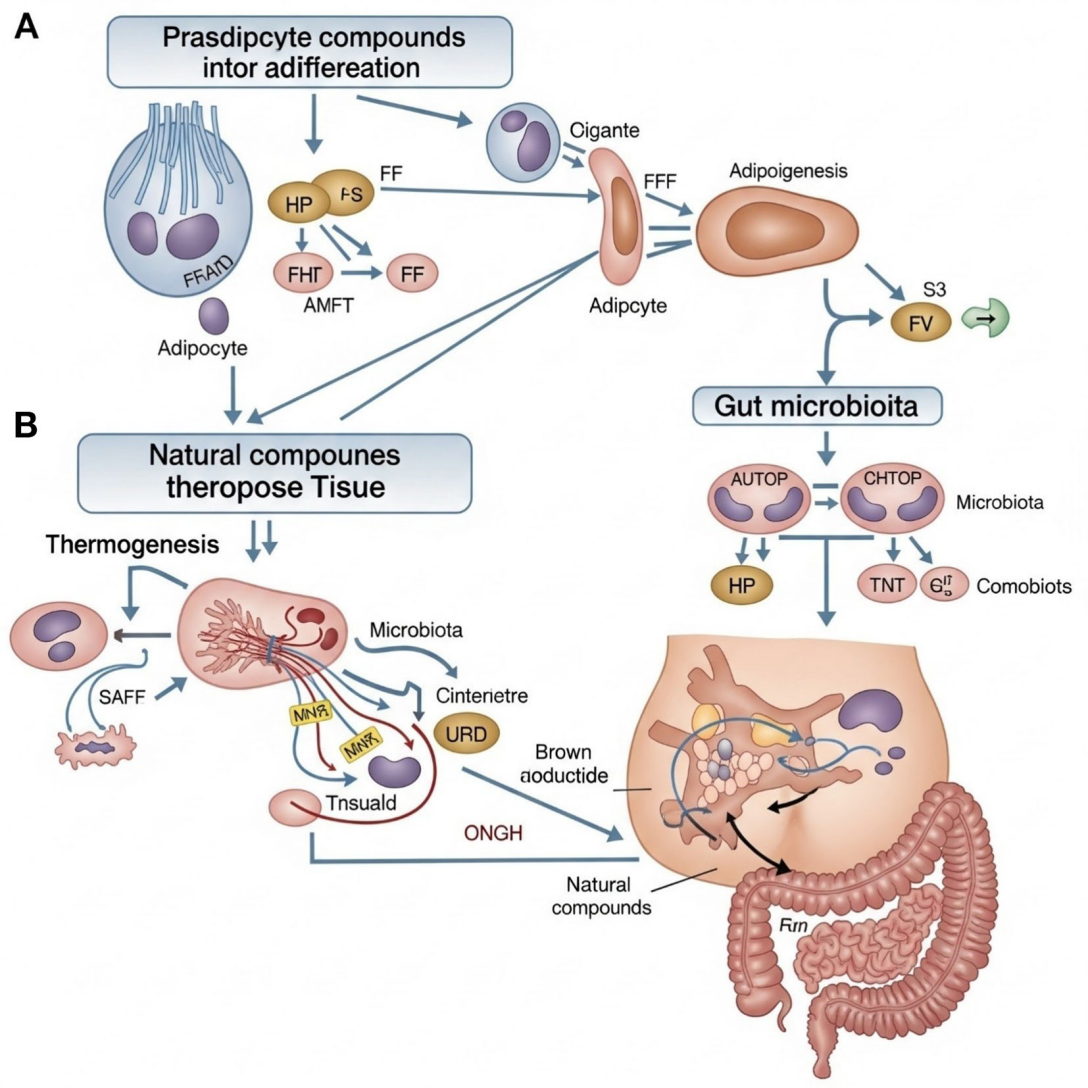
Interactions of natural compounds with metabolic pathways influencing three key biological processes: adipogenesis, thermogenesis, and the development of gut microbiota. The image is described in detail to understand the intricate details. It was divided into two main panels: **(A, B)**, each focusing on different aspects while showing some interconnectedness. **(A)** Natural compounds and adipogenesis and preadipocyte differentiation: The left side of **(A)** illustrates the process of adipogenesis. It starts with a “Preadipocyte” (pre-adipocyte), which is an immature fat cell. The arrows show its transformation into “mature adipocytes”, which are fully developed fat cells capable of storing lipids. Influence of natural compounds: The arrows originating from “Natural compounds” point toward various stages and components involved in pre-adipocyte differentiation and adipogenesis. This signifies that natural compounds can modulate this process. Some arrows point to elements labeled “FR”, “F”, “T”, “P”, “FFF”, “TVS”, and “G2”. These likely represent specific signaling pathways, transcription factors, or molecular targets that natural compounds interact with to either promote or inhibit adipocyte maturation. Adipogenesis pathway: From “mature adipocytes”, the arrows lead to “adipogenesis”, indicating the overall process of fat cell formation and growth. Further cellular components like “NC OP” and “CFF” are shown, suggesting downstream effects or different types of fat cells that can form. Gut microgenesis (within **(A)**): This sub-section, while seemingly separate, might imply an initial or parallel process related to the gut or its cellular components. Elements like “GS”, “SDGO”, “ND CNP”, “RS”, and “FV” could represent specific molecules or cellular events involved in gut development or early metabolic programming that might indirectly link to adipogenesis. **(B)** Natural compounds, thermogenesis, and microbiota interaction natural compounds and thermogenesis (brown adipose tissue): The top left of **(B)** focuses on thermogenesis, specifically involving “brown adipose tissue” (BAT). “Preait” (likely pre-adipocytes or progenitor cells for BAT) are shown, leading to mature BAT cells. “Natural compounds” directly influence BAT, with arrows indicating their role in regulating its activity. “Signat regulate comfustion” (likely “Signals regulate combustion”) highlights the thermogenic function of BAT, where it burns fat to produce heat. “TNF” (tumor necrosis factor), a pro-inflammatory cytokine, is shown in relation to BAT, suggesting its involvement in the thermogenic process or its modulation by natural compounds. Microbiota and metabolic interplay: The right side and bottom of **(B)** illustrate the crucial role of the gut microbiota and its interactions with host metabolism. A simplified diagram of the human gut shows the location of the “Microbiota”. The arrows from “Microbiota” point to various labels like “GF”, “FD”, “Suard”, “Alard”, and “Meat” (which is likely a misspelling or abbreviation for “metabolites” or “metabolism”). This indicates that the gut bacteria produce various compounds or signals that influence host physiology. These microbial products or signals then interact with other components like “Adpama” (potentially related to adiponectin or other adipokines) and influence “TNF” and “Tamr CNB” (likely a specific signaling pathway or a complex involved in metabolism). Ultimately, these interactions loop back to influence the “Microbiota&” and the gut environment, creating a complex feedback loop that impacts overall host metabolism, including processes related to fat storage (adipogenesis) and energy expenditure (thermogenesis).

The various pathways as mentioned below (**Figure 2**) were taken as examples where natural compounds play an important role by specific mechanisms.

### Short-chain fatty acid (SCFA) production

Short-chain fatty acid (SCFA) production plays a beneficial role in butyrate, propionate, and acetate to improve insulin sensitivity and reduce inflammation (43). There are few natural prebiotics like inulin and oligofructose (chicory root, garlic) which promote SCFA-producing bacteria (e.g., *Bifidobacterium* spp.). In bile acid metabolism, the bile acids also activate FXR and TGR5 receptors, influencing lipid metabolism (44). The few detailed natural modulators as exemplified here, like chitosan (crustacean shells), are used to bind bile acids, reducing fat absorption. Many such examples can likewise be explored in this regard.

### Clinical evidence and therapeutic applications

Current research underscores the significant role of plant-derived bioactive compounds in combating obesity through microbiome regulation and multi-system mechanisms as shown in **Table 4**. Clinical trials reveal that polyphenolic compounds, particularly resveratrol (500 mg daily) and green tea catechins (300–400 mg daily), effectively boost the populations of beneficial gut bacteria (*Akkermansia* and *Bifidobacterium*), resulting in improved metabolic function and measurable fat reduction (4% to 5% decrease) (45). The plant alkaloid berberine (500 mg three times daily) demonstrates remarkable efficacy in rebalancing gut flora and stimulating metabolic regulators. Dietary fiber interventions (15 g/day) promote the growth of beneficial microbes (*Roseburia* and *Faecalibacterium*), correlating with a significant weight reduction (6.2%). Curcumin formulations (1 g/day) exhibit novel effects on appetite regulation through gut–brain communication pathways. These natural agents operate through multiple biological pathways, influencing metabolic signaling, inflammatory responses, and energy regulation. The latest clinical guidelines advocate for customized treatment approaches combining these compounds with probiotics. While showing great promise, further research is required to establish optimal treatment protocols and verify long-term benefits. Advanced microbiome analysis now facilitates tailored therapeutic strategies, positioning these natural solutions as innovative options in obesity care.

**Table 4:** Key studies on plant-derived bioactive compounds to combat obesity.

Compound	Dose	Model	Outcome	References
Resveratrol	500 mg daily	Human clinical trials	Boosts the populations of beneficial gut bacteria (*Akkermansia* and *Bifidobacterium*) and improves metabolic function, resulting in 4% to 5% fat reduction	(47)
Green tea catechins	300–400 mg daily	Human clinical trials	Boosts the populations of beneficial gut bacteria (*Akkermansia* and *Bifidobacterium*) and improves metabolic function, resulting in 4% to 5% fat reduction	(48)
Berberine	500 mg three times daily	Human clinical trials	Rebalances gut flora and stimulates metabolic regulators, promoting a significant weight loss	(49)
Dietary fiber	15 g/day	Human clinical trials	Promotes the growth of beneficial microbes (*Roseburia* and *Faecalibacterium*), resulting in 6.2% weight reduction	(50)
Curcumin	1 g/day	Human clinical trials	Exhibits novel effects on appetite regulation through gut–brain communication pathways	(25)

**Figure 3:**
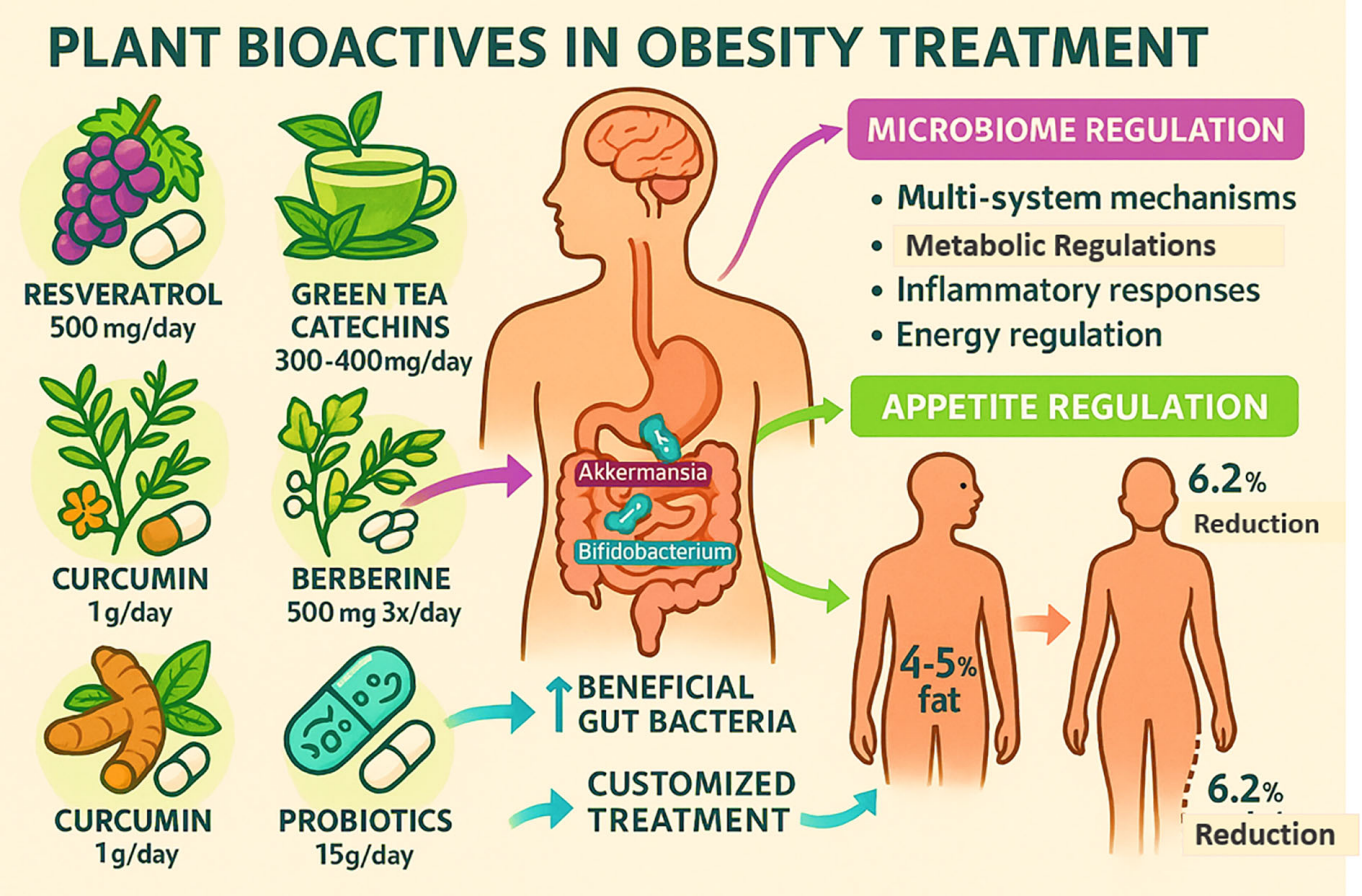
Impact of natural compounds in obesity treatment.

With a focus on appetite regulation and the microbiome, **Figure 3** depicts the role of probiotics and a number of plant bioactive in the treatment of obesity. It highlights certain natural substances that have been shown in scientific studies to have anti-obesity effects: berberine (500 mg three times a day), curcumin (1 g daily), green tea catechins (300–400 mg daily), and resveratrol (500 mg daily). Probiotics (15 g/day) are also included because of their beneficial effects on gut health (18, 47, 49, 50). By supporting good gut flora like *Akkermansia* and *Bifidobacterium*, which have been shown to have a positive effect on inflammation and metabolic health, these bioactives help people manage their weight. These drugs promote improvements in gut microbiota that result in better treatment outcomes through mechanisms involving multi-system effects, metabolic regulation, inflammatory response reduction, and appetite control. According to the figure, clinically, these interventions can result in 4% to 5% decrease in body fat and 6.2% decrease in total weight. Combining probiotics and plant bioactives offers a promising, multifaceted strategy to manage obesity that supports energy regulation and individualized care based on the makeup of the gut microbiota (49, 50).

## Challenges and future perspectives

### Bioavailability and formulation

Many natural compounds (e.g., curcumin, resveratrol) suffer from poor absorption. The probable solutions are nanoencapsulation and phospholipid complexes (e.g., phytosomes). Even studies on synergistic combinations—for example, green tea extract (EGCG + caffeine)—show better efficacy than single compounds.

### Precision nutrition approaches

This refers to the sector of personalized interventions based on gut microbiome profiling and genetic polymorphisms (e.g., FTO gene variants). *In vivo* studies investigating natural compounds for obesity-related disorders reveal complex interactions. Various compounds often work synergistically, where their combined effect is greater than the sum of individual effects, offering multi-targeted approaches to combat obesity (51)—for instance, combinations of polyphenols like epigallocatechin-3-gallate (EGCG) and caffeine have shown enhanced anti-obesity synergy by modulating gut microbiota and bile acid metabolism (52). Conversely, antagonistic interactions can occur, where one compound diminishes the efficacy of another, potentially compromising therapeutic outcomes. Understanding these intricate dynamics is crucial for developing effective and safe natural product-based strategies for obesity management.

## Conclusions

Obesity presents a multifaceted global health crisis that demands innovative treatment approaches. Naturally derived bioactive compounds from plant, fruit, and marine sources have emerged as promising therapeutic agents due to their ability to influence critical metabolic processes involved in weight regulation (53, 54). These substances—including polyphenols like resveratrol, alkaloids such as berberine, and marine carotenoids—exert their anti-obesity effects through multiple mechanisms. They modulate adipocyte differentiation, enhance fat breakdown, stimulate thermogenic activity, and favorably alter gut microbiota composition. Clinical studies have validated their potential, showing significant improvements in body composition and metabolic parameters. However, several limitations must be addressed, including variable bioavailability between individuals and the need for optimized delivery systems. Cutting-edge solutions like nano-formulations and personalized nutrition strategies based on microbial and genetic profiling offer exciting possibilities to enhance therapeutic outcomes. Moving forward, research should prioritize establishing standardized protocols, investigating long-term effects, and developing synergistic combinations. By bridging traditional wisdom with contemporary scientific validation, these natural compounds could revolutionize obesity management by providing safer, multi-targeted alternatives to conventional treatments. Their integration into comprehensive lifestyle interventions may offer sustainable solutions to this pervasive health challenge. Future research on the metabolic regulation of obesity by natural compounds should align with emerging policy guidelines, such as the WHO’s Global Action Plan on obesity and the FDA’s framework for natural product evaluation. Encouraging open-access data sharing (per NIH and EU Horizon Europe mandates) and multidisciplinary collaboration will accelerate mechanistic insights and therapeutic validation. Policymakers must integrate preclinical findings into regulatory pathways for nutraceuticals, ensuring safety and efficacy. Public–private partnerships, as promoted by initiatives like the UN Decade of Action on Nutrition, can translate research into affordable, evidence-based interventions, addressing obesity as a global health priority.
